# Development and validation of an interpretable machine learning-based predictive model for breast cancer bone metastasis

**DOI:** 10.3389/fonc.2026.1692909

**Published:** 2026-07-03

**Authors:** Caiyun Fan, Ming Tian, Zhendong Ding, Jun Peng, Gulisitan Yiliyiming, Mingjiang Fan, Abuduaini Tuerxun, Binxu Qiu, Xiaojuan Zhu

**Affiliations:** 1 Department of Anesthesiology, The First People’s Hospital of Kashi, Kashi, China; 2 Faculty of Data Science, City University of Macau, Macao, Macao SAR, China; 3 Postdoctoral Station of Pharmacy, the Third Xiangya Hospital, Central South University, Changsha, China; 4 Department of Anesthesiology, Memorial Hospital, Sun Yat-sen University, Guangzhou, China; 5 Department of Breast and Thyroid Surgery, The First People’s Hospital of Kashi, Kashi, China; 6 Breast Center, West China Hospital, Sichuan University, Chengdu, China

**Keywords:** bone, breast cancer, interpretable, machine learning, metastasis

## Abstract

**Background:**

Breast cancer is one of the most common malignancies worldwide, with bone metastasis representing its most frequent distant metastatic form, significantly worsening patient prognosis. This study aims to develop a machine learning-based predictive model for accurately assessing the risk of bone metastasis in breast cancer patients, thereby enabling personalized risk stratification, early clinical intervention, and optimized treatment strategies.

**Methods:**

This study utilized the Surveillance, Epidemiology, and End Results database as the primary data source to develop machine learning models for predicting bone metastasis risk in breast cancer patients. Initially, univariate and multivariate logistic regression analyses were conducted to screen key predictive variables; subsequently, eight machine learning algorithms were constructed based on the screening results.10-fold cross-validation employed for hyperparameter optimization. Following training, model performance was evaluated on an internal test cohort and externally validated on 342 real-world cases from an independent hospital cohort. Model assessment incorporated multiple metrics, including area under the curve (AUC), area under the precision-recall curve (AUPRC), decision curve analysis, and calibration curves. Additionally, SHAP analysis was applied to enhance model interpretability, and a web-based calculator was developed based on the optimal model to facilitate clinical application and decision support.

**Results:**

Baseline characteristics across cohorts indicated that the majority of patients were aged over 50 years, female, predominantly with the HR+/HER2− molecular subtype, and exhibited a low incidence of bone metastasis. Univariate and multivariate logistic regression analyses identified key independent risk factors, including age >50 years, higher tumor grade, advanced T stage, N stage, clinical stage and HR-/HER2- subtype factors included radiotherapy, surgery, and married status. The LGB model demonstrated superior performance, achieving an AUC of 0.98 in the training set and 10-fold cross-validation (standard deviation=0.00), 0.98 in the internal validation set, and 0.91 in the external validation set; AUPRC values across the three cohorts were 0.96, 0.79, and 0.87, respectively; decision curve analysis showed excellent net clinical benefit within the 0.1-0.8 threshold range; calibration curves further confirmed high concordance between predicted probabilities and actual event rates. SHAP analysis highlighted surgery as the primary protective factor, followed by N stage, T stage, and radiotherapy as risk enhancers; for example, advanced N stage was associated with positive SHAP values, indicating a significant increase in bone metastasis risk.

**Conclusions:**

This study developed an interpretable LGB model accompanied by a web-based calculator, thereby advancing personalized risk stratification, early detection of bone metastasis and optimized treatment strategies.

## Introduction

Breast cancer (BC) remains one of the most prevalent malignancies worldwide, with recent epidemiological data indicating a steady rise in incidence rates (1). In 2025, an estimated 316,950 new cases of invasive BC are projected among women in the United States alone, alongside 59,080 cases of ductal carcinoma *in situ*, reflecting a 1% annual increase over the past decade, particularly among younger women and those with higher body mass indices (2). Globally, the age-standardized incidence rate of breast cancer was approximately 47.8 cases per 100,000 women in 2022, with incidence rates increasing by 1-5% annually in many high-income countries, underscoring the growing public health burden (3). Bone metastasis represents a common and devastating complication, occurring in up to 70% of patients with advanced BC (4), with incidence rates ranging from 3.6% at initial diagnosis to 13.6% within 15 years for early-stage cases (5). Early prediction of bone metastasis is critical, as it enables timely interventions that can improve survival, reduce skeletal-related events such as fractures and pain, and enhance quality of life by facilitating targeted therapies like bone-modifying agents (6). This study addresses the research question of whether machine learning models can accurately predict bone metastasis risk in BC patients, hypothesizing that integrating diverse clinical features through advanced algorithms will outperform traditional methods in prognostic accuracy (7).

Despite substantial advances in BC research, existing predictive models for bone metastasis continue to exhibit important methodological and clinical limitations. Early predictive efforts primarily relied on traditional statistical approaches, particularly logistic regression models incorporating demographic and clinicopathological variables such as age, tumor grade, tumor size, and hormone receptor status. Although these models offer interpretability, they generally assume linearity and independence among predictors, limiting their ability to capture the complex, non-linear biological interactions underlying metastatic progression. As a result, their predictive accuracy and risk stratification performance remain suboptimal, especially in the context of high-dimensional clinical data (8). In recent years, machine learning–based models, including random forests, support vector machines, and gradient boosting algorithms, have been increasingly applied to predict bone metastasis in BC patients. These approaches demonstrate improved discrimination by modeling non-linear relationships and interactions among variables. However, a substantial proportion of published models are developed exclusively using public databases such as SEER, which may introduce selection bias and restrict population heterogeneity. Consequently, the generalizability and external validity of these models are often limited, as they may not adequately reflect real-world clinical diversity in patient characteristics, treatment strategies, and healthcare systems (9). Moreover, few studies incorporate independent external cohorts or cross-regional validation, further constraining clinical translation. The lack of integration of multi-institutional data and real-world clinical settings may lead to models that perform well in retrospective analyses but fail to maintain robustness across different clinical environments (10).

To address these gaps, the Surveillance, Epidemiology, and End Results (SEER) database offers substantial advantages, including comprehensive population-based data on cancer incidence, survival, and treatment for nearly 50% of the U.S. population, enabling robust analysis of large cohorts with long-term follow-up (11, 12). Machine learning further enhances prediction by automating feature selection, handling non-linear relationships, and improving accuracy through techniques like ensemble methods, which have demonstrated 15-25% better performance in cancer prognosis compared to conventional statistics (13).

In this study, we utilize SEER data to train multiple machine learning models for bone metastasis prediction, validate them externally using data from an independent hospital cohort, and select the optimal algorithm based on performance metrics. Additionally, we develop a web-based calculator to aid clinical decision-making. The subsequent sections outline the methodology, present results including model comparisons and validation, and discuss implications for personalized BC management.

## Method

### Study population and data selection

This retrospective study utilized data from the SEER database (2010-2022) and a Chinese medical institution. The SEER program, maintained by the U.S. national cancer institute, collects population-based cancer incidence and survival data from multiple registries that collectively cover approximately 48% of the U.S. population, ensuring strong representativeness and data reliability. Given that no personally identifiable information was used, patient informed consent and institutional review board approval were not required for this retrospective analysis. Breast cancer cases were identified using the International Classification of Diseases for Oncology, Third Edition (ICD-O-3) primary site codes C50.0–C50.9 and histological codes 8500–8543, which correspond to the main invasive breast carcinoma subtypes according to the world health organization (WHO) classification of tumors of the breast, 5th edition (2019). Patients diagnosed with breast cancer were extracted from the SEER database and subsequently divided into training and internal validation cohorts using a 7:3 ratio. For the Chinese cohort, data were retrospectively collected from the electronic medical records of the First People’s Hospital of Kashi between 2015 and 2023. Inclusion criteria were: (1) pathologically confirmed primary breast cancer; (2) complete clinicopathological and follow-up information; and (3) absence of other concurrent malignancies. Exclusion criteria included: (1) incomplete clinical or follow-up data; (2) diagnosis based solely on imaging without histological confirmation; and (3) presence of another primary cancer. All patient data were manually reviewed by two independent investigators to ensure accuracy and consistency. Clinical variables collected from the Chinese cohort included age, sex, tumor location, histological grade, TNM stage, molecular subtype, treatment modalities (surgery, radiotherapy, and chemotherapy), and bone metastasis status. Data extraction and cleaning were performed following standardized procedures to ensure consistency with the variable definitions used in the SEER database. Missing or ambiguous values were verified through cross-checking with pathology reports and follow-up records. To maintain consistency with SEER database classification, patients from the Chinese medical institution were categorized as “Other” under the race variable. Treatment modalities (surgery, radiotherapy, and chemotherapy) were dichotomized as either “yes” or “no” according to treatment administration status. This study was approved by the ethics committee of the first people’s hospital of Kashi (No. (2025)-47). All research was conducted in accordance with relevant guidelines/regulations and the Declaration of Helsinki, and informed consent was obtained from all participants.

### Machine learning model construction and evaluation

Univariate and multivariate logistic regression analyses were employed to evaluate risk factors associated with bone metastasis in the training cohort (14). Odds ratios (ORs) with 95% confidence intervals (CIs) were calculated, with OR > 1 indicating potential risk factors. Variables that demonstrated statistical significance in the multivariate logistic regression analysis were subsequently incorporated as input features for machine learning model development. The machine learning methodology was implemented using Python (version 3.10) and the scikit-learn library (version 0.24). The dataset was randomly partitioned into training and testing sets at a ratio of 7:3. Eight machine learning algorithms were utilized for model construction: Logistic Regression (LR), Gradient Boosting Machine (GBM), Extreme Gradient Boosting (XGB), Random Forest (RF), Light Gradient Boosting Machine (LGB), Decision Tree (DT), and Naive Bayes (BNB). We conducted 10-fold cross-validation on the developed algorithm, while employing random search for hyperparameter optimization. The predictive performance of each model was comprehensively evaluated on the testing set using multiple metrics: area under the receiver operating characteristic curve (AUC), area under the precision-recall curve (AUPRC), decision curve analysis (DCA), and calibration curves. This multi-faceted evaluation approach enabled robust assessment of model discrimination, precision, clinical utility, and calibration.

### Explainable machine learning methods

To enhance the interpretability of the machine learning model, we applied the Shapley Additive Explanations (SHAP) method, a game theory–based algorithm that quantifies the contribution of each variable to the model’s output (14). SHAP values were computed to determine the relative importance and direction of influence of each feature in the prediction process. The analysis was performed using the SHAP package in Python, generating global and individual-level visualizations of feature importance and interaction effects. This approach allowed for transparent assessment of model behavior and feature relevance across the dataset.

### Statistical analysis

Data processing and statistical analyses were conducted using R software (version 4.0.5). Continuous variables were presented as median with interquartile range, while categorical variables were expressed as frequencies and percentages. For comparative analyses, continuous variables were evaluated using independent samples t-tests, and categorical variables were assessed using chi-square tests. All statistical tests were two-sided, with P<0.05 considered statistically significant. This analytical approach was selected to provide clinically meaningful insights into the relationships between independent and dependent variables within our study population.

## Result

### Baseline characteristics

The baseline characteristics of breast cancer patients in the training set (N = 151,662), internal validation set (N = 64,999), and external validation set (N = 342) are summarized in **Table 1**. Across all cohorts, the majority of patients were aged over 50 years, and females accounted for more than 99% of cases. Radiation therapy was administered to approximately half of the patients in the training and internal validation sets, but was slightly more common in the external set. Chemotherapy receipt rates were 39.2% and 39.5% in the training and internal validation sets, respectively, but markedly lower at 21.3% in the external set. Surgical intervention was performed in the vast majority of patients across cohorts. Histological grade II was the most prevalent. Stage I disease was the most frequent in the training and internal validation sets, whereas stage III was more common in the external set. The HR+/HER2- molecular subtype was the most common, while HER2 positivity was lower in the external set. The overall incidence of bone metastasis was low. The source code for this study has been made publicly available in **Supplementary Material 1**.

**Table 1:** Baseline characteristics of breast cancer patients in training, internal validation, and external validation cohorts.

Variables	Training	Internal validation (test)	External validation
	N=151662	N=64999	N=342
Age			
≤50	30162 (19.9%)	12960 (19.9%)	9 (2.63%)
>50	121500 (80.1%)	52039 (80.1%)	333 (97.4%)
Sex			
Male	1046 (0.69%)	474 (0.73%)	1 (0.29%)
Female	150616 (99.3%)	64525 (99.3%)	341 (99.7%)
Literal			
Biliter	19 (0.01%)	4 (0.01%)	0 (0)
Right	76883 (50.7%)	32816 (50.5%)	161 (47.1%)
Left	74760 (49.3%)	32179 (49.5%)	181 (52.9%)
Race			
White	103616 (68.3%)	44737 (68.8%)	0 (0.0%)
Black	10885 (7.18%)	4554 (7.01%)	0 (0.0%)
Others	37161 (24.5%)	15708 (24.2%)	342 (100.0%)
Radiation			
No	74685 (49.2%)	32095 (49.4%)	148 (43.3%)
Yes	76977 (50.8%)	32904 (50.6%)	194 (56.7%)
Chemotherapy			
No	91792 (60.5%)	39524 (60.8%)	269 (78.7%)
Yes	59870 (39.5%)	25475 (39.2%)	73 (21.3%)
Marriage			
No	67209 (44.3%)	28925 (44.5%)	170 (49.7%)
Yes	84453 (55.7%)	36074 (55.5%)	172 (50.3%)
Surgery			
No	7715 (5.09%)	3233 (4.97%)	22 (6.43%)
Yes	143947 (94.9%)	61766 (95.0%)	320 (93.6%)
Grade			
I	38754 (25.6%)	16734 (25.7%)	108 (31.6%)
II	67295 (44.4%)	28844 (44.4%)	163 (47.7%)
III	45514 (30.0%)	19381 (29.8%)	71 (20.8%)
IV	99 (0.07%)	40 (0.06%)	0 (0)
T			
T1	92267 (60.8%)	39716 (61.1%)	220 (64.3%)
T2	46593 (30.7%)	19851 (30.5%)	97 (28.4%)
T3	8605 (5.67%)	3680 (5.66%)	12 (3.51%)
T4	4197 (2.77%)	1752 (2.70%)	13 (3.80%)
N			
N0	106069 (69.9%)	45572 (70.1%)	277 (81.0%)
SN1	33806 (22.3%)	14434 (22.2%)	48 (14.0%)
N2	7339 (4.84%)	3076 (4.73%)	10 (2.92%)
N3	4448 (2.93%)	1917 (2.95%)	7 (2.05%)
Stage			
Stage I	79946 (52.7%)	34540 (53.1%)	128 (37.4%)
Stage II	51611 (34.0%)	21952 (33.8%)	19 (5.56%)
Stage III	15349 (10.1%)	6488 (9.98%)	195 (57.0%)
Stage IV	4756 (3.14%)	2019 (3.11%)	0 (0)
Breast-subtype			
HR-/HER2-	15991 (10.5%)	6870 (10.6%)	29 (8.48%)
HR-/HER2+	6256 (4.12%)	2576 (3.96%)	7 (2.05%)
HR+/HER2-	114185 (75.3%)	49072 (75.5%)	288 (84.2%)
HR+/HER2+	15230 (10.0%)	6481 (9.97%)	18 (5.26%)
ER			
Negative	23595 (15.6%)	10053 (15.5%)	36 (10.5%)
Positive	128067 (84.4%)	54946 (84.5%)	306 (89.5%)
PR:			
Negative	39709 (26.2%)	16891 (26.0%)	88 (25.7%)
Positive	111953 (73.8%)	48108 (74.0%)	254 (74.3%)
HER2:			
Negative	130176 (85.8%)	55942 (86.1%)	317 (92.7%)
Positive	21486 (14.2%)	9057 (13.9%)	25 (7.31%)
Bone-met			
No	148633 (98.0%)	63693 (98.0%)	322 (94.2%)
Yes	3029 (2.00%)	1306 (2.01%)	20 (5.85%)

### Univariate and multivariate logistic regression analysis

In univariate logistic regression analysis, factors significantly associated with an increased risk of bone metastasis in patients with breast cancer included age >50 years, chemotherapy, higher grade, advanced T stage, lymph node involvement, advanced stage, HR-/HER2- subtype, and HER2 positivity (*P* < 0.001). Protective factors comprised male sex, radiotherapy, marital status, surgery, and PR positivity (*P* < 0.001). Laterality and race showed no significant associations (*P* > 0.05). In multivariate logistic regression analysis, independent risk factors included age >50 years (OR: 1.63, 95% CI 1.46 - 1.83, *P* < 0.001), marital status (OR: 1.39, 95% CI 1.28–1.51, *P* < 0.001), advanced T stage (T4: OR: 4.96, 95% CI 4.06-5.85, *P* < 0.001), lymph node involvement (N3: OR: 6.34, 95% CI 5.41-7.44, *P* < 0.001), advanced stage (stage IV: OR: 3.20, 95% CI 3.00–3.45, *P* < 0.001), and HR+/HER2+ subtype (OR: 2.29, 95% CI 1.92-2.73, *P < 0.001*). Protective factors comprised radiotherapy (OR: 0.15, 95% CI 0.13-0.17, *P* < 0.001) and surgery (OR: 0.03, 95% CI 0.03–0.04, *P* < 0.001). Gender, chemotherapy, race, and PR status showed no significant associations (*P* > 0.05) (**Table 2**).

**Table 2:** Univariate and multivariate logistic regression analysis of risk factors for bone metastasis in breast cancer patients.

Variable	*P*	OR (95%CI)	*P*	OR (95%CI)
Age				
≤50		1.00 (Reference)		1.00 (Reference)
>50	<.001	1.45 (1.33 ~ 1.58)	<.001	1.63 (1.46 ~ 1.83)
Sex				
Male		1.00 (Reference)		
Female	0.814	0.96 (0.67 ~ 1.37)		
Literal				
Biliter		1.00 (Reference)		
Right	0.057	0.51 (0.07 ~ 1.05)		
Left	0.058	0.51 (0.07 ~ 1.08)		
Race				
White		1.00 (Reference)		
Black	0.367	1.05 (0.95 ~ 1.16)		
Others	0.090	0.94 (0.87 ~ 1.01)		
Radiation				
No		1.00 (Reference)		1.00 (Reference)
Yes	<.001	0.74 (0.70 ~ 0.79)	<.001	0.15 (0.13 ~ 0.17)
Chemotherapy				
No		1.00 (Reference)		
Yes	0.059	1.07 (0.88 ~ 1.26)		
Marriage				
No		1.00 (Reference)		1.00 (Reference)
Yes	<.001	0.85 (0.80 ~ 0.90)	<.001	1.39 (1.28 ~ 1.51)
Surgery				
No		1.00 (Reference)		1.00 (Reference)
Yes	<.001	0.02 (0.02 ~ 0.03)	<.001	0.03 (0.03 ~ 0.04)
Grade				
I		1.00 (Reference)		
II	0.124	1.79(0.84 ~ 2.74)		
III	0.057	1.04 (0.96 ~ 1.12)		
IV	0.157	1.82 (0.85 ~ 3.68)		
T				
T1		1.00 (Reference)		1.00 (Reference)
T2	<.001	5.03 (4.60 ~ 5.50)	<.001	2.44 (2.15 ~ 2.73)
T3	<.001	13.99 (12.62 ~ 15.51)	<.001	3.52 (2.74 ~ 4.30)
T4	<.001	45.46 (41.16 ~ 50.20)	<.001	4.96 (4.06~ 5.85)
N				
N0		1.00 (Reference)		1.00 (Reference)
N1	<.001	6.04 (5.60 ~ 6.50)	<.001	3.93 (3.52 ~ 4.38)
N2	<.001	7.41 (6.67 ~ 8.23)	<.001	4.91 (4.17 ~ 5.79)
N3	<.001	15.70 (14.20 ~ 17.35)	<.001	6.34 (5.41 ~ 7.44)
Stage				
Stage I		1.00 (Reference)		1.00 (Reference)
Stage II	<.001	1.67 (1.49 ~ 1.88)	<.001	1.10 1.03 ~ 1.17)
Stage III	<.001	7.86 (7.06 ~ 8.76)	<.001	2.35 (2.05 ~ 2.65)
Stage IV	<.001	24.98 (22.69 ~ 27.50)	<.001	3.20 (3.00 ~ 3.45)
Breast-subtype				
HR-/HER2-		1.00 (Reference)		1.00 (Reference)
HR-/HER2+	<.001	1.74 (1.48 ~ 2.05)	0.171	1.17 (0.94 ~ 1.45)
HR+/HER2-	0.027	1.13 (1.01 ~ 1.26)	<.001	1.92 (1.65 ~ 2.24)
HR+/HER2+	<.001	1.94 (1.71 ~ 2.20)	<.001	2.29 (1.92 ~ 2.73)
ER				
Negative		1.00 (Reference)		
Positive	0.665	1.02 (0.94 ~ 1.11)		
PR:				
Negative		1.00 (Reference)		
Positive	0.576	0.98 (0.92 ~ 1.05)		
HER2:				
Negative		1.00 (Reference)		1.00 (Reference)
Positive	0.029	1.07 (1.01 ~ 1.14)	<.001	0.46 (0.32 ~ 0.60)

### Machine learning model construction

**Figure 1:**
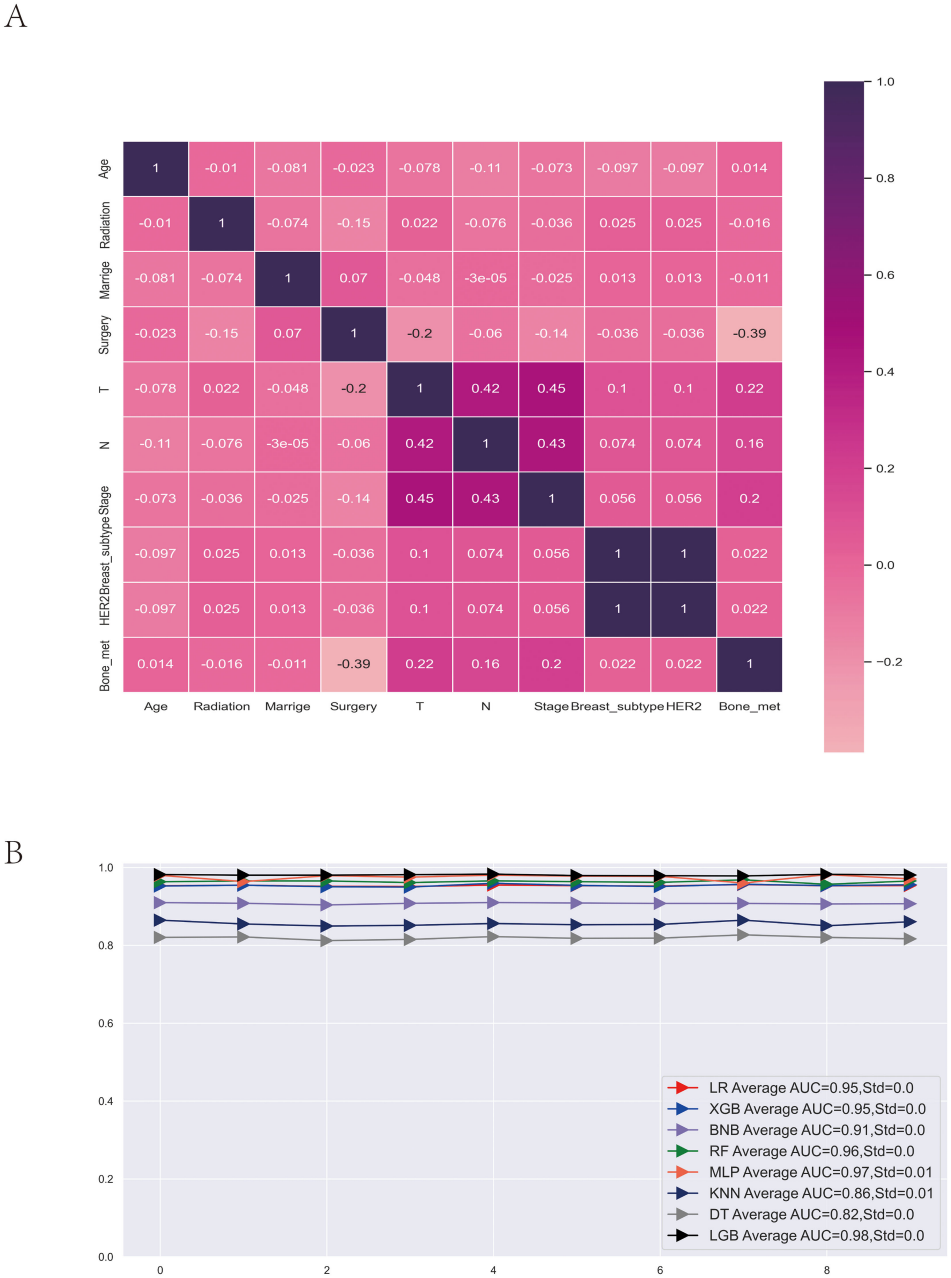
Correlation matrix of clinical variables and comparative performance of eight machine learning algorithms with 10-fold cross-validation for predicting bone metastasis in breast cancer. **(A)** Pearson correlation matrix illustrating the pairwise correlations among clinical and pathological variables. **(B)** AUC values for eight machine learning algorithms obtained through 10-fold cross-validation. ROC, receiver operating characteristic curve; AUC, average area under the curve.

**Figure 2:**
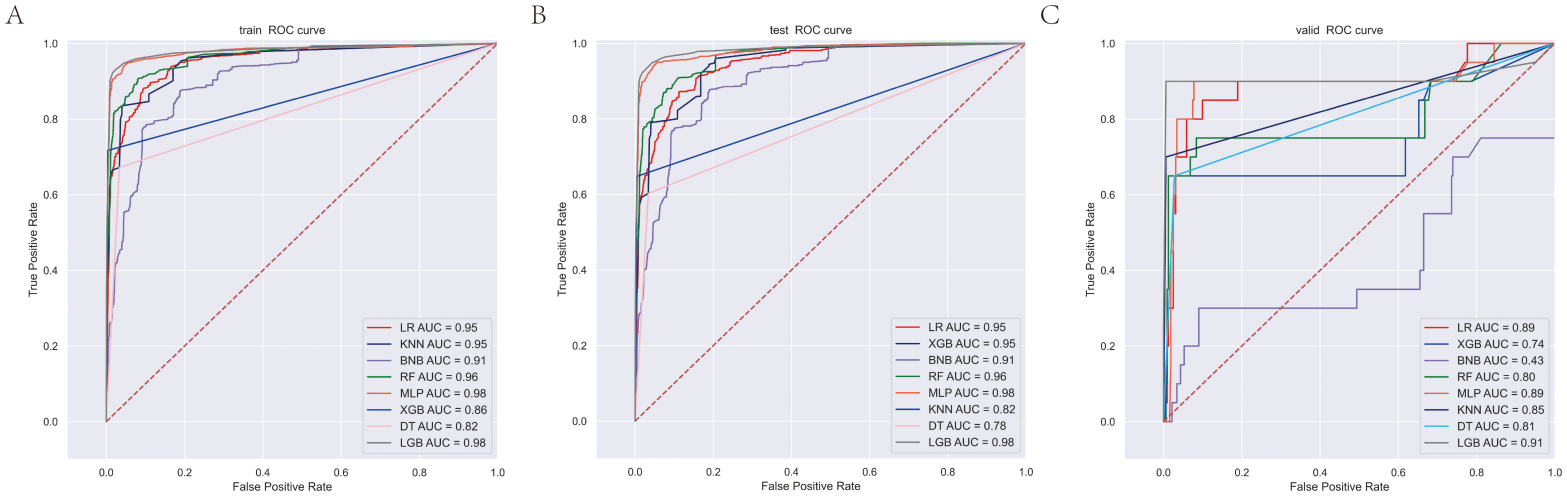
ROC curves of the eight model for predicting bone metastasis in breast cancer across training **(A)**, internal validation **(B)**, and external validation cohorts **(C)**. ROC, receiver operating characteristic curve.

**Figure 3:**
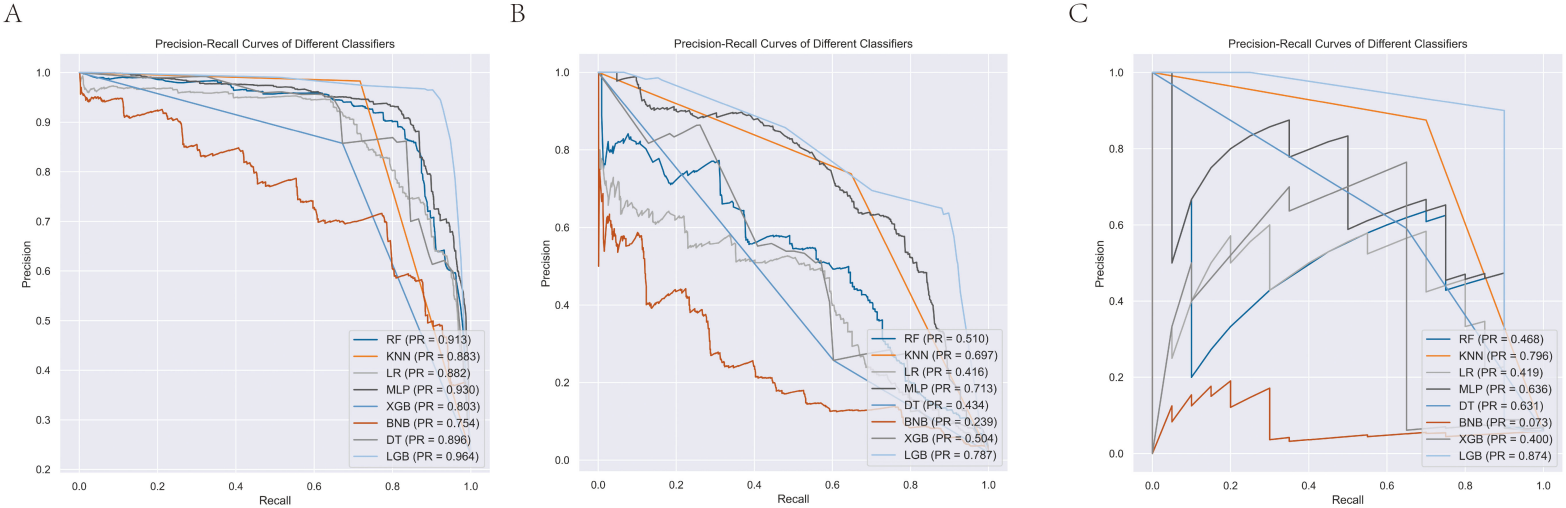
PR curves of the eight model for predicting bone metastasis in breast cancer across training **(A)**, internal validation **(B)**, and external validation cohorts **(C)**. PR, precision-recall curve.

**Figure 4:**
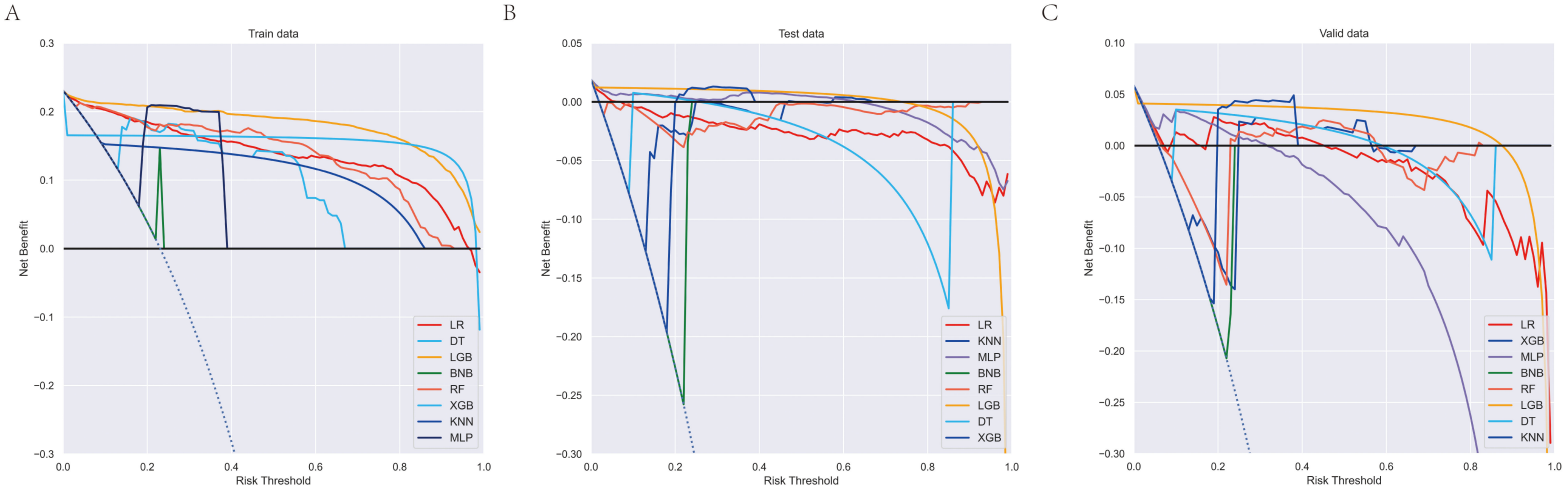
DCA curves of the eight model for predicting bone metastasis in breast cancer across training **(A)**, internal validation **(B)**, and external validation cohorts **(C)**. DCA, decision curve analysis.

**Figure 5:**
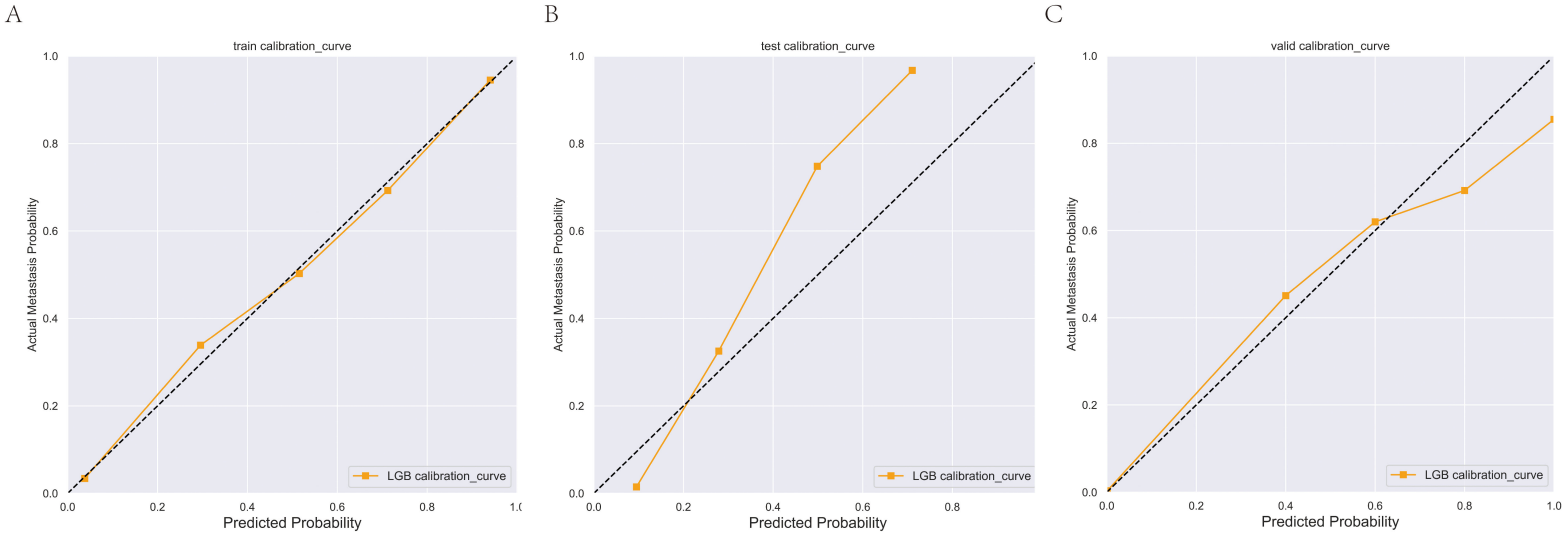
Calibration curves of the XGBoost model for predicting bone metastasis in breast cancer across training **(A)**, internal validation **(B)**, and external validation cohorts **(C)**.

To develop a robust predictive model for bone metastasis risk in breast cancer patients, we employed 10-fold cross-validation for hyperparameter optimization. In the 10-fold cross-validation on the training set, the LGB model demonstrated the highest AUC value (AUC = 0.98, SD = 0.00) (**Figure 1B**). ROC analysis revealed that all models exhibited high discriminative performance on the training set, with AUC values ranging from 0.82 (DT) to 0.98 (LGB). A similar trend was observed on the test set, where AUC values varied from 0.78 (DT) to 0.98 (LGB) (**Figures 2A–C**). However, performance attenuated on the external validation set, with LGB achieving the highest AUC of 0.91. The PR curves highlighted LGB’s superiority in the precision-recall trade-off, with an average precision score of 0.96 on the training set, and slightly lower scores on the test and validation sets (0.79 and 0.87, respectively) (**Figures 3A–C**). In the training and test sets, LGB consistently yielded the highest net benefit across threshold ranges of 0.1 to 0.8. On the validation set, LGB maintained superior net benefit at clinically relevant thresholds (0.2-0.6), underscoring its utility in decision-making for high-risk patients (**Figures 4A–C**). Calibration curves indicated good agreement between predicted probabilities and observed outcomes for LGB across the training, test, and validation sets (**Figures 5A–C**). Based on its superior AUC, AUPRC, net benefit, and calibration performance across all datasets, particularly in the external validation set, LGB was selected as the optimal mod**el.**

### Interpretation of machine learning models

**Figure 6:**
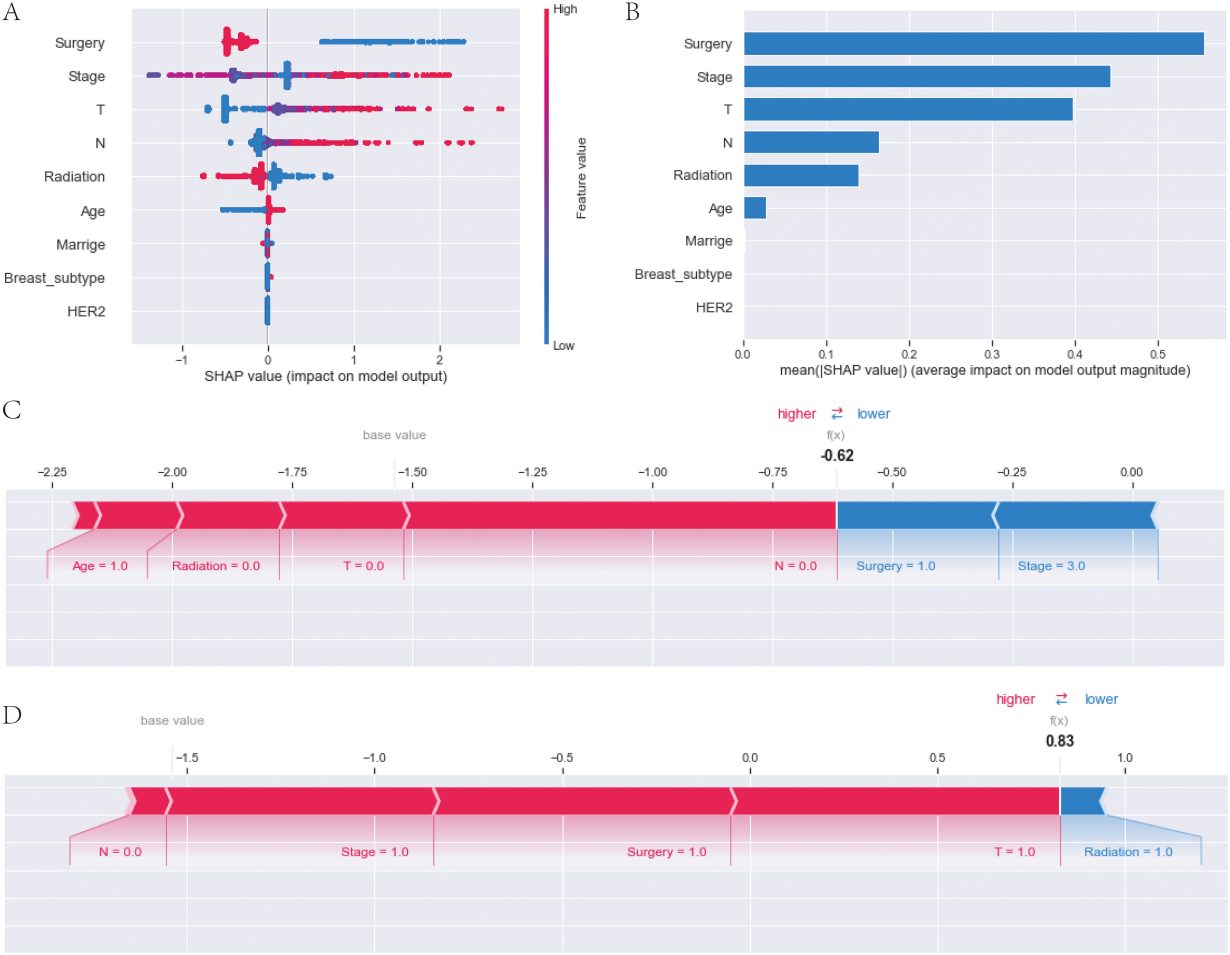
SHAP analysis of feature importance and individual prediction explanations in the breast cancer bone metastasis prediction model. **(A)** SHAP summary plot showing the contribution of each clinical feature to the model output. **(B)** Mean absolute SHAP values ranking the overall importance of features in predicting bone metastasis. **(C, D)** SHAP force plots illustrating the contribution of individual features to the predicted risk for two representative patients.

Due to the black-box nature of the LGB model, we employed the SHAP method to enhance its interpretability. **Figure 6** presents the SHAP summary plot for the LGB model, illustrating the relative importance of surgery-related features in predicting the risk of bone metastasis in breast cancer patients. In this plot, each dot represents a single prediction for an individual patient, with the color indicating the magnitude of the corresponding feature value, and the x-axis denoting the SHAP value, which reflects the direction and magnitude of the feature’s impact on the prediction outcome. Notably, whether surgery was performed exerts the most significant influence on the model’s predictions. For instance, features such as N stage, T stage, and radiotherapy rank prominently in the SHAP value ordering, underscoring their strong predictive capability in distinguishing the occurrence of bone metastasis. Specifically, advanced N stages are typically associated with positive SHAP values, suggesting a potential link to elevated bone metastasis risk; in contrast, radiotherapy correlates with negative SHAP values, implying a possible protective effect against distant metastasis. Overall, the SHAP analysis not only improves the model’s transparency but also provides quantitative insights for identifying high-risk populations for postoperative bone metastasis. In clinical practice, this interpretability enhances the model’s trustworthiness and applicability, particularly in scenarios requiring clear delineation of risk factors and decision-making support.

## Discussion

This study leveraged large-scale data from the SEER database to develop machine learning models, which were subsequently validated using real-world data to confirm their accuracy. Furthermore, we created a web-based calculator based on the final model, facilitating its clinical dissemination and enabling personalized decision-making for patients. The findings revealed significant associations between bone metastasis in breast cancer patients and several key clinical and demographic factors, including advanced age, marital status, surgical intervention, radiotherapy, breast cancer subtype, HER2 status, clinical staging, T staging, and N staging. These associations are consistent with established biological mechanisms and epidemiological patterns in breast cancer progression, thereby providing a robust foundation for enhancing risk stratification and tailoring individualized treatment strategies.

Advanced age emerges as a prominent risk factor for bone metastasis, with underlying mechanisms attributable to age-related declines in immune surveillance, hormonal imbalances, and the accumulation of genetic mutations-all of which facilitate metastatic dissemination (15, 16). This observation aligns with the multi-step carcinogenesis model, wherein prolonged exposure to carcinogenic stressors heightens the likelihood of epithelial-mesenchymal transition and seeds metastatic cells within the bone microenvironment, particularly in patients over 50 years old (17, 18). Furthermore, older patients often exhibit bone remodeling alterations driven by the senescence-associated secretory phenotype, which amplifies the RANKL/OPG signaling pathway to create a fertile niche for tumor cell colonization (19). Marital status, especially being unmarried or divorced, correlates with an elevated risk of bone metastasis (20–22). This association may operate through psychosocial mechanisms, such as chronic stress-induced cortisol elevation, which suppresses immune function and promotes tumor progression via the NF-κB pathway. According to the biopsychosocial model of cancer outcomes, marital support can buffer depression and enhance adherence to screening and treatment, thereby reducing the metastatic burden. In contrast, social isolation exacerbates inflammation and angiogenesis, fostering distant spread (23, 24). Overall, this underscores the role of social environmental factors in modulating epigenetic changes that influence metastasis.

Surgical interventions exhibit a protective effect against bone metastasis, likely by excising the primary tumor mass and disrupting the formation of pre-metastatic niches through reduced release of circulating tumor cells and cytokines (25, 26). According to Paget’s “seed and soil” hypothesis, resection of the primary lesion interrupts vascular and lymphatic dissemination pathways while achieving superior local control to prevent epithelial-mesenchymal transition induction. Similarly, radiotherapy’s association with lower metastatic risk stems from its induction of DNA damage in residual tumor cells, activation of anti-tumor immunity via immunogenic cell death, and alteration of the bone extracellular matrix to inhibit osteoclast-mediated homing (27). These therapies align with the oligometastasis theory, wherein localized treatments can eradicate micrometastases before they become clinically apparent.

Breast cancer subtypes exhibit distinct risks, with hormone receptor-positive subtypes demonstrating higher bone tropism due to estrogen-driven osteoclast activation and CXCL12/CXCR4 chemotaxis in the bone marrow (28). This is explained by Perou and Sorlie’s intrinsic subtype model, which differentiates subtypes based on gene expression profiles: luminal subtypes favor osteolytic metastases through PTHrP secretion, whereas triple-negative basal-like subtypes, while less bone-specific, are overall more aggressive. HER2 status further modulates this dynamic, as HER2-overexpressing tumors display enhanced invasiveness via PI3K/AKT pathway activation, promoting epithelial-mesenchymal transition and vascular endothelial growth factor-mediated angiogenesis, thereby facilitating bone colonization. Targeted therapies like trastuzumab disrupt this process by inhibiting HER2 dimerization, reducing metastatic potential in line with signal transduction models (29).

Advanced clinical staging, along with higher T and N stages, is strongly associated with bone metastasis (30, 31). This association mirrors the sequential progression outlined in the Halstedian paradigm, where larger primary tumors and lymph node involvement signal increased genomic instability and clonal evolution toward enhanced metastatic capability. From a mechanistic perspective, elevated T staging correlates with hypoxia-induced HIF-1α expression, which upregulates genes involved in invasion and bone mimicry, whereas N staging indicates lymphatic spread that seeds distant sites through tumor-draining lymph nodes (32). These elements are incorporated into the TNM staging system, which forecasts outcomes based on anatomical extent and biological aggressiveness (33).

Given the strong clinical and biological relevance of these staging-related variables, the model’s high discriminative performance is understandable. However, the AUC values approaching 0.98 should still be interpreted with caution, as such high estimates may partly reflect overfitting or dataset-specific patterns. Moreover, discrimination alone does not establish clinical utility. Variables such as surgery and radiotherapy should not be interpreted as causal protective factors against bone metastasis, since they may reflect earlier disease stage, lower tumor burden, or treatment selection bias. Therefore, the model should be regarded primarily as a tool for risk stratification rather than causal inference or direct treatment decision-making. Furthermore, patient-centered outcomes, particularly patient-reported outcomes such as symptom burden, quality of life, functional status, and treatment-related experiences, were not included in the present model, although these indicators are increasingly recognized as important evidence for oncology decision-making (34).

Regarding model performance, LGB outperforms other algorithms in predicting the risk of bone metastasis. This superiority can be attributed to its gradient boosting framework (35). Unlike simpler models, LGB incorporates regularization to prevent overfitting, automatic feature selection through histogram-based algorithms and exclusive feature bundling, as well as ensemble learning that aggregates weak learners to achieve better generalization (36, 37).

## Limitation

Nevertheless, several limitations of this study should be acknowledged. First, the SEER database may introduce selection and information bias due to the absence or incomplete recording of important clinical variables, which may affect model accuracy. Second, although real-world data were used for external validation, the limited sample size and single-institution origin may restrict the generalizability of the findings. In addition, regional differences in healthcare systems and treatment patterns may influence patient characteristics. Therefore, despite the good performance observed across cohorts, application of this model in other clinical settings should be interpreted cautiously.

## Data Availability

The original contributions presented in the study are included in the article/**SupplementaryMaterial**. Further inquiries can be directed to the corresponding author.
